# Health and wellbeing needs and priorities in mining host communities in South Africa: a mixed-methods approach for identifying key SDG3 targets

**DOI:** 10.1186/s12889-021-12348-6

**Published:** 2022-01-11

**Authors:** Brian Rice, Delia Boccia, Daniel J. Carter, Renay Weiner, Lebohang Letsela, Mariken de Wit, Rebecca Pursell, Michael Jana, Ana Maria Buller, Mitzy Gafos

**Affiliations:** 1 London School of Hygiene & Tropical Medicine, 15-17 Tavistock Place, London, WC1H 9SH UK; 2 Research and Training for Health and Development, Johannesburg, South Africa; 3 School of Public Health, University of the Witwatersrand, Johannesburg, South Africa; 4 Soul City Institute for Social Justice, Johannesburg, South Africa; 5 School of Social Sciences, University of the Witwatersrand, Johannesburg, South Africa

**Keywords:** SDG, South Africa, Mixed-methods, HIV, TB, Substance abuse, Road traffic accidents

## Abstract

**Background:**

The global mining industry has an opportunity to mobilize resources to advance progress against the Sustainable Development Goals (SDGs). In 2018, the Anglo-American Group outlined aspirations for mining host communities to meet the SDG3 health targets. To progress from aspiration to action we designed and implemented a mixed-methods approach to attain a deeper understanding of the health and wellbeing priorities within the local context of host communities of fifteen mines in South Africa.

**Methods:**

To identify local needs and priorities relating to SDG3 targets in host communities, stakeholder workshops and key informant interviews were conducted between June and August 2019. A baseline assessment of health data, related to each of the SDG3 targets and indicators and to each host community location, was also conducted. Findings emerging from the qualitative and quantitative baseline assessments were compared to identify the extent to which health issues aligned and health and wellbeing priority areas for action.

**Results:**

A total of 407 people participated in the workshops, and 85 key informants were interviewed. Quantitative data were available at sub-national level for seven of the nine SDG3 targets and eleven of the 21 indicators. Key priority areas for action identified through alignment of the qualitative and quantitative data were maternal mortality (SDG3.1), HIV (SDG3.3.1), tuberculosis (SDG3.3.2), substance abuse (SDG3.5), and road traffic accidents (SDG3.6) We found consistency in the individual, interpersonal, community, societal, and structural factors underlying these priority areas. At a structural level, poor access to quality healthcare was raised at every workshop as a key factor underlying the achievement of all SDG3 targets. Of the five priority areas identified, HIV, TB and substance abuse were found to overlap in the study communities in terms of risk, burden, and underlying factors.

**Conclusions:**

We demonstrate a mixed method approach for identifying local health needs and prioritised SDG3 targets in mining host communities. Consistency in reporting suggests the need for effective, efficient and feasible interventions to address five priority areas. Given the prominent economic role of the mining sector in South Africa, it can play a critical role in implementing programmatic activities that further progress towards achieving the SDG3 targets.

## Background

In 2015, United Nations member states adopted the 2030 Agenda for Sustainable Development and its seventeen Sustainable Development Goals (SDGs) [1]. These interrelated goals aim to reduce poverty, improve health and education, protect the planet, and ensure all people enjoy peace and prosperity.

In 2016, members of the United Nations Development Programme and World Economic Forum identified the global mining industry as having an unprecedented opportunity to mobilize resources to advance the SDGs, given their activities are often located in remote and less-developed areas, and can, when managed correctly, bring investment and infrastructure at scale [2]. Responding to the SDGs also presented the mining industry with an opportunity to mitigate some of the adverse health impacts associated with their activities [3]. In 2017, the authors of a study reflecting on opportunities to contribute to SDGs, recommended that research and practitioner communities explore how national-level SDGs can be translated into organisation-level indicators for mining companies in sub-Saharan Africa to respond to through direct action at the local level [4].

.In recognition of the role mining companies could play in advancing progress against the SDGs, in 2018, the Anglo American Group published their Sustainable Mining Plan [5]. Under the broad objective of building thriving communities, three key goals relating to education, livelihoods and health and wellbeing were laid out. The health and wellbeing goal aligns with SDG3.1 to 3.9 targets. The first milestone of this goal was to establish baseline assessments at every site so that priority SDG targets could be identified. To deliver against this milestone, the Sustainable Development Goals Health and Wellbeing consortium at the London School of Hygiene and Tropical Medicine was established to design and implement a mixed-methods research study in South Africa to identify local health needs and prioritise SDG3 targets for intervention. Across South Africa, mining host communities are estimated to be home to 5.4 million people [6].

## Methods

An assessment of the health and wellbeing priorities in the host communities of fifteen Anglo American mining operations across four provinces in South Africa was conducted using a mixed-methods approach where existing health data were quantitatively appraised and qualitative research was conducted. Following discussions with mining stakeholders, a host community was defined as being within at least fifty kilometres of a mining operation. All host communities within this range were included regardless of their relationship with mining operations (for example, regardless as to whether the majority or all residents were non-workforce). As our focus is on the independent health needs of each community, we do not consider the type of mining operation in our analysis.

**Fig. 1:**
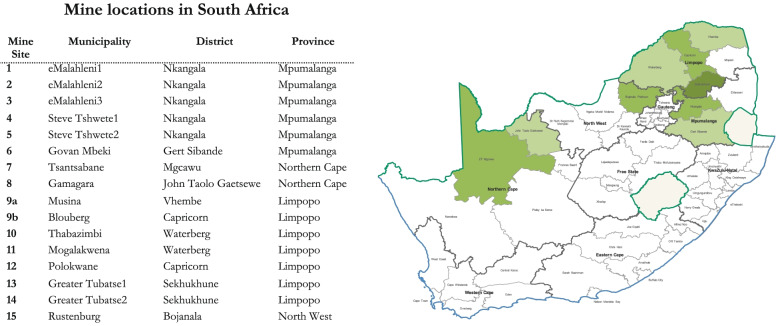
Mine locations in South Africa

The fifteen mining operations in South Africa are located in the provinces of Mpumalanga, Northern Cape, Limpopo, and North West, and all host communities identified were open towns (i.e. not company towns). Of the fifteen operations, seven mine platinum, two mine iron ore, and six mine coal. Figure 1 presents the province, municipality and district where each mine site is located. For reasons of confidentiality, we have masked the names of each mine site.

### Qualitative appraisal of local needs and priorities

Stakeholder workshops and key informant interviews were conducted between June and August 2019, where participants were encouraged to identify local needs and priorities, relating to SDG3 targets, in their host communities. Study participants were identified and invited to volunteer to participate by mine social-performance teams based on their community stakeholder fora, other stakeholders, and/or by the South African research team (consisting of consortium members at London School of Hygiene and Tropical Medicine, Soul City Institute for Social Justice, and Research and Training for Health and Development). The study was explained to participants using a Participant Information Sheet (specific to workshops or interviews) and all participants provided written informed consent.

#### Workshops

A workshop guide was developed using a participatory action research approach that included techniques such as listing, scoring, ranking, and group discussions [7]. The one-day workshops were conducted at community venues centrally located within a host community and comprised participant-led activities where participants identified which SDG3 targets were local priorities by indicating them on SDG-specific wall posters. After reaching consensus on the three or four priority SDG3 targets, break-out groups were formed to discuss each in turn; feedback was then provided to the whole group and suggestions for action put forward. Findings were recorded on record sheets, and these sheets were reviewed and subjected to quality control by the study team. At each site, at least one of the facilitators were fluent in the local languages. In Mpumalanga province, municipality-wide workshops, instead of single-site workshops, were conducted due to the mining operations interacting with the same body of stakeholders (i.e. one workshop was conducted for the three mine sites located in eMalahleni municipality, and one conducted for the two mine sites located in Steve Tshwete). In Limpopo, workshops were conducted in two municipalities serving one mine site (i.e. Blouberg and Musina).

#### Key informant interviews

We developed key informant interview guides for stakeholders from local community-based organisations (such as women’s and youth groups, non-government organisations, schoolteachers, and health service providers) and official stakeholders (including local, regional, national level representatives of the Ministry of Health, mine staff, and public health service facility managers). The interview guides were semi-structured and covered organisation focus, participant’s experience and involvement in health and wellbeing improvement, key considerations for achieving SDG targets, priority areas of action, gaps in the response, and suggestions for action. Findings were recorded on a record sheet and reviewed by the study team.

#### Analytical framework

A framework to support a thematic analysis of the data was developed and reviewed. Qualitative data arising from each workshop and key informant interview were analysed separately and then combined for each mine or district. Using the analysis framework, the following topics were recorded: data overview and sources, SDG target prioritisation, underlying factors, and suggestions for action. Guided by the socio-ecological model, participant responses were categorised to identity structural, societal, community, and individual-level underlying factors relating to each target in order to explore the interdependence between multiple behavioural and social determinants [8].

### Quantitative appraisal of health data

In relation to the location of each mine, we undertook a baseline assessment of health data related to each of the SDG3 targets and indicators. To facilitate a comparison of the quantitative data (collected in 2019) with qualitative data, we focused on the most recent year data were available (i.e. we do not consider multiple time points).

The assessment commenced with a review of the SDGs United Nations Indicators website, and province, district and municipal data available from governmental and non-governmental reports [9]. The major sources drawn upon for this activity were the District Health Plans and District Health Barometers, supplemented by data from Arrive Alive for road traffic accidents (RTAs) and the National Statistics Service, including the latest national mortality report for South Africa [10]. A non-systematic review of the relevant published literature was also conducted. Municipality was the default geography for results. Where information was not available at the municipality level we defer, depending on data availability, to district or province. In 2020, a second round of quantitative data collection was conducted in relation to HIV so that newly published numbers from the 2019 Thembisa model could be included [11, 12].

### Identifying health and wellbeing priorities

An SDG indicator was identified as a priority area when it had been put forward for selection by multiple stakeholders in workshops and/or interviews and/or when the quantitative baseline data indicated a clear burden of disease in the community above the national or the designated SDG targets. As described above, during the qualitative appraisal, participants (individually or in groups) identified three or four priority areas for action in their community. These expressed priorities were then collectively counted and ranked. In the quantitative assessment, health and wellbeing indicators were priority ranked based on the extent to which their burden exceeded their respective target. Following this process of ranking, the qualitative and quantitative priorities were compared and assessed for alignment.

The study protocol was approved by the Human Sciences Research Council research ethics committee in South Africa (ref: 10/20/02/19) and the London School of Hygiene and Tropical Medicine research ethics committee in the United Kingdom (ref: 16349).

## Results

### Qualitative assessment

A total of 407 participants were included in the workshops; 64% of participants were female, 37% were from community-based organisations, 29% were healthcare providers, 15% worked in government departments, and 19% worked in other types of organisations, such as traditional authorities, police forums, and youth forums. There were 31 participants on average per workshop (range: 12-56). A total of 85 key informants were interviewed, of whom 55% were female, 44% were mine representatives, 19% were workshop attendees, and 38% were other identified stakeholders who had not attended a workshop. Of the 48 stakeholders who were not mine employees, 50% were from the health sector, 27% from community-based organisations, 17% from government departments, and 6% from other organisations.

There was a good level of consistency across stakeholders participating in the qualitative assessment, with three key targets emerging for prioritisation: SDG3.3 (communicable diseases), SDG3.5 (substance abuse), and SDG3.6 (RTAs). SDG3.9 (air pollution) was also frequently raised as a possible key target. Table 1 presents prioritised targets based on citation frequency during the workshops and interviews conducted between June and August 2019.

**Table 1:**
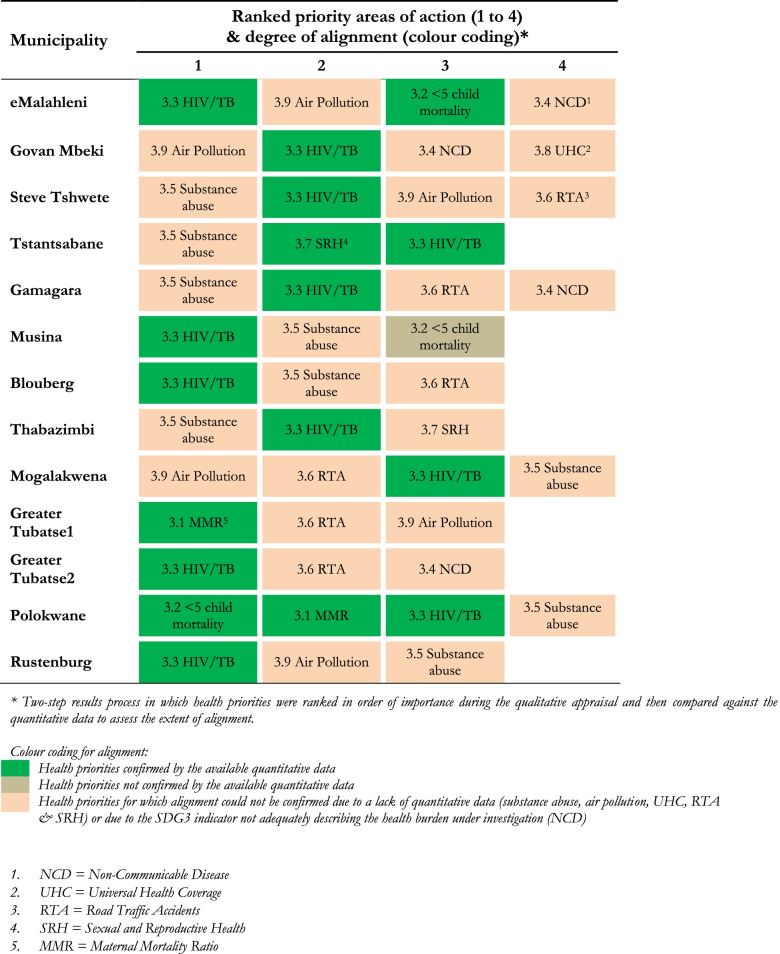
Priority areas of action identified during workshops and interview, and degree of alignment with quantitative data

**Fig. 2:**
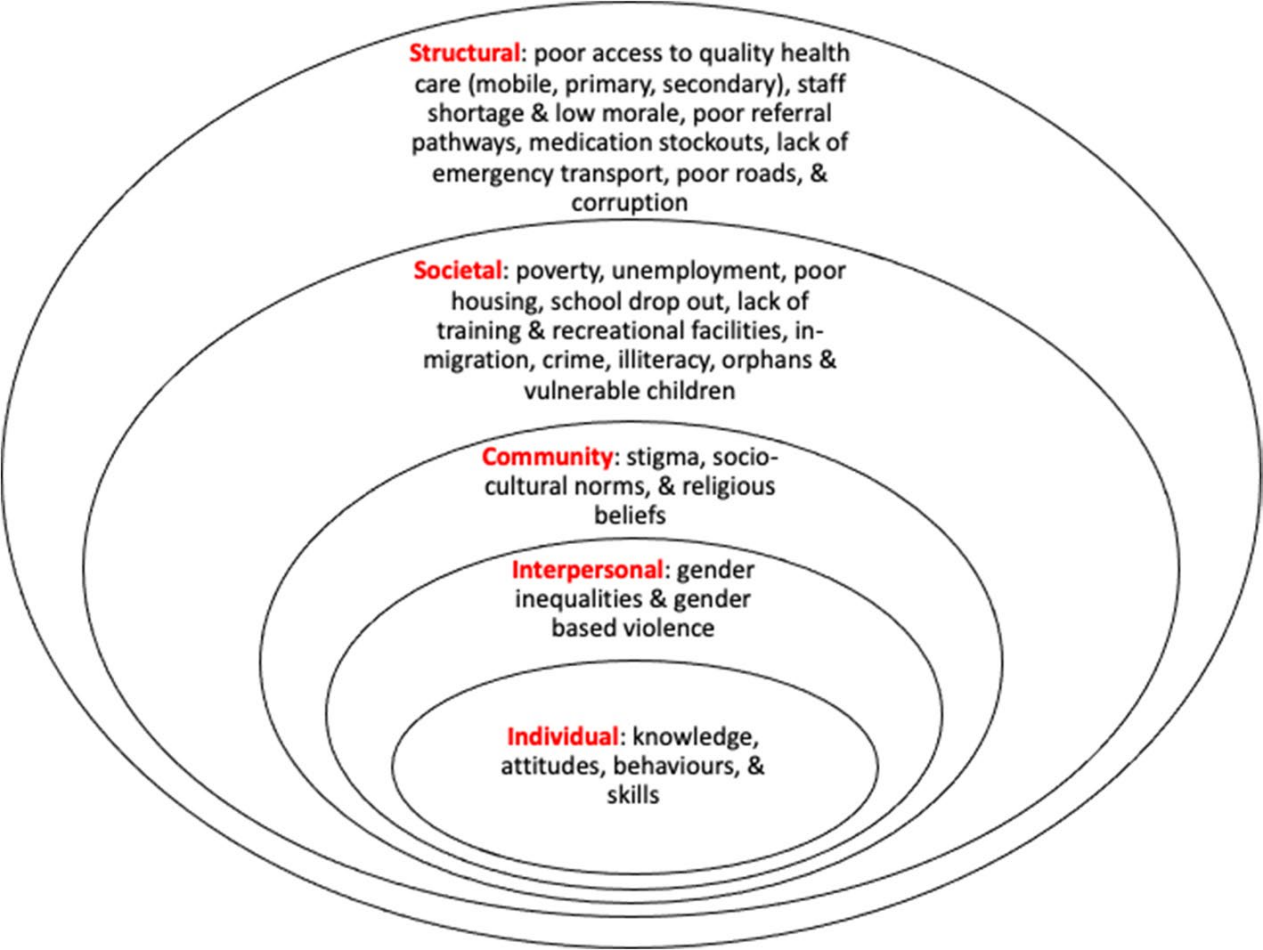
Socioeconomic framework demonstrating different levels of factors influencing health and wellbeing common to all study sites

There was a good degree of consistency across workshops and interviews in the reported individual, interpersonal, cultural, societal, and structural factors underlying poor health and wellbeing. It was of interest that despite SDG 3.8 (universal health coverage) only being listed as a priority in the Govan Mbeki workshop, the need for ‘*access to quality essential health-care services and access to […] essential medicines*’ was raised as a key factor underlying the achievement of SDG3 targets in all workshops. Figure 2 presents a summary of the main factors influencing poor health and wellbeing across the communities.

### Quantitative assessment

Quantitative data were available at provincial, district or municipal level for seven of the nine SDG3 targets (3.1 to 3.7), and eleven of the 21 SDG3 indicators.^1^ The quantitative assessment generally aligned with the priorities identified in the qualitative data, demonstrating a high burden of HIV and TB (SDG3.3), RTA-related mortality (SDG3.6), and alcohol consumption (SDG3.5). The assessment also highlighted substantial maternal mortality (SDG3.1). Progress towards reducing communicable disease burden (SDG3.3) and maternal mortality (SDG3.1) was found to be poor in most communities. Results arising from the quantitative assessment are presented in detail below.

### Qualitative and quantitative assessment alignment

Table 1 presents the degree of alignment between the health priorities identified in the qualitative assessment and the available quantitative data. Although alignment for substance abuse and RTAs could not be confirmed due to insufficient local data, all four provinces were shown to have levels of alcohol consumption above the national average, as well as a higher than average mortality burden due to RTAs. The alignment process for air pollution could not be conducted due to an absence of quantitative data.

**Fig. 3:**
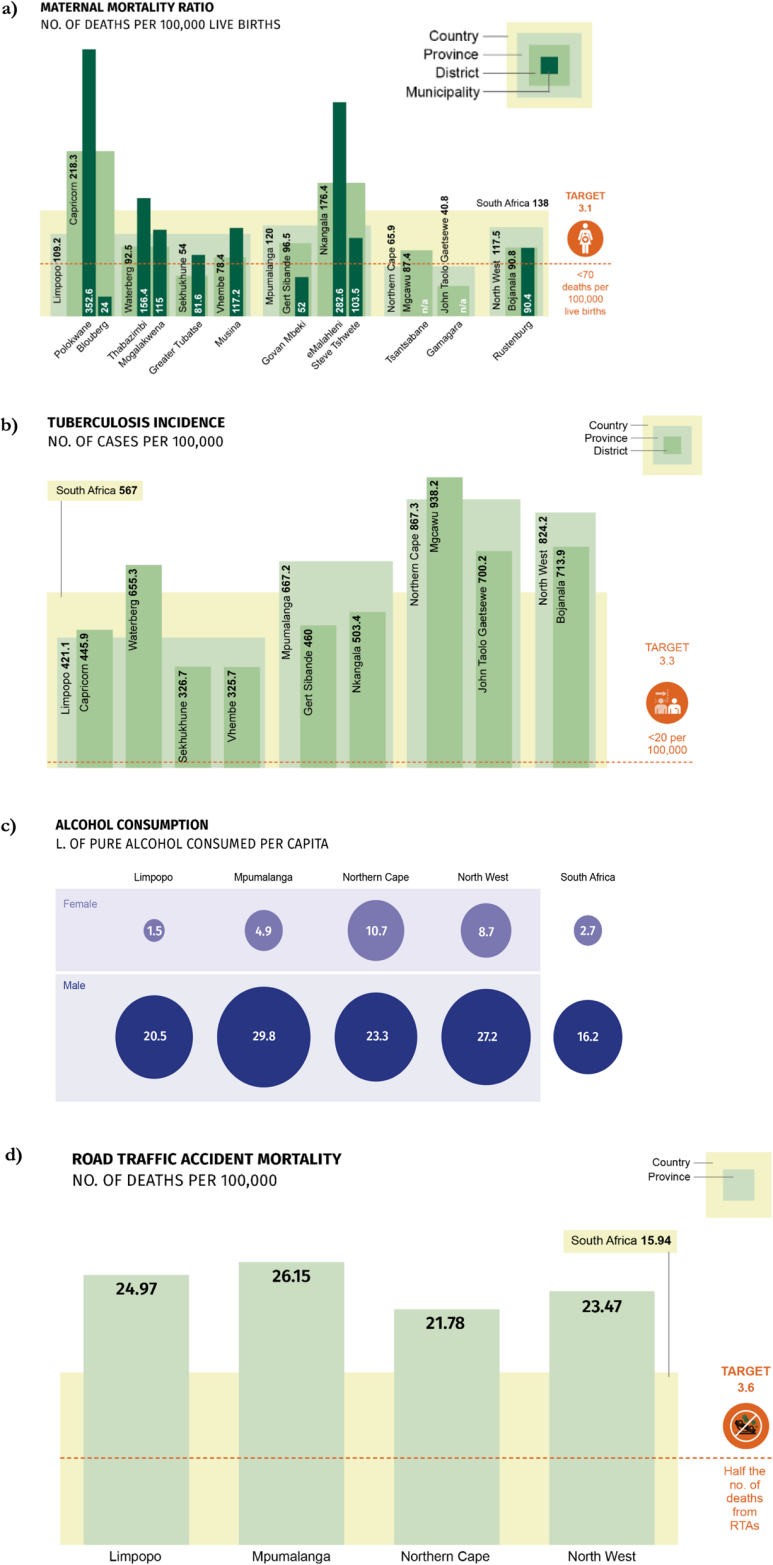
Priority SDG3 indicators 3.1 (maternal mortality), 3.3.2 (TB), 3.5 (substance abuse) and 3.6 (RTAs) by national and sub-national geographies

**Fig. 4:**
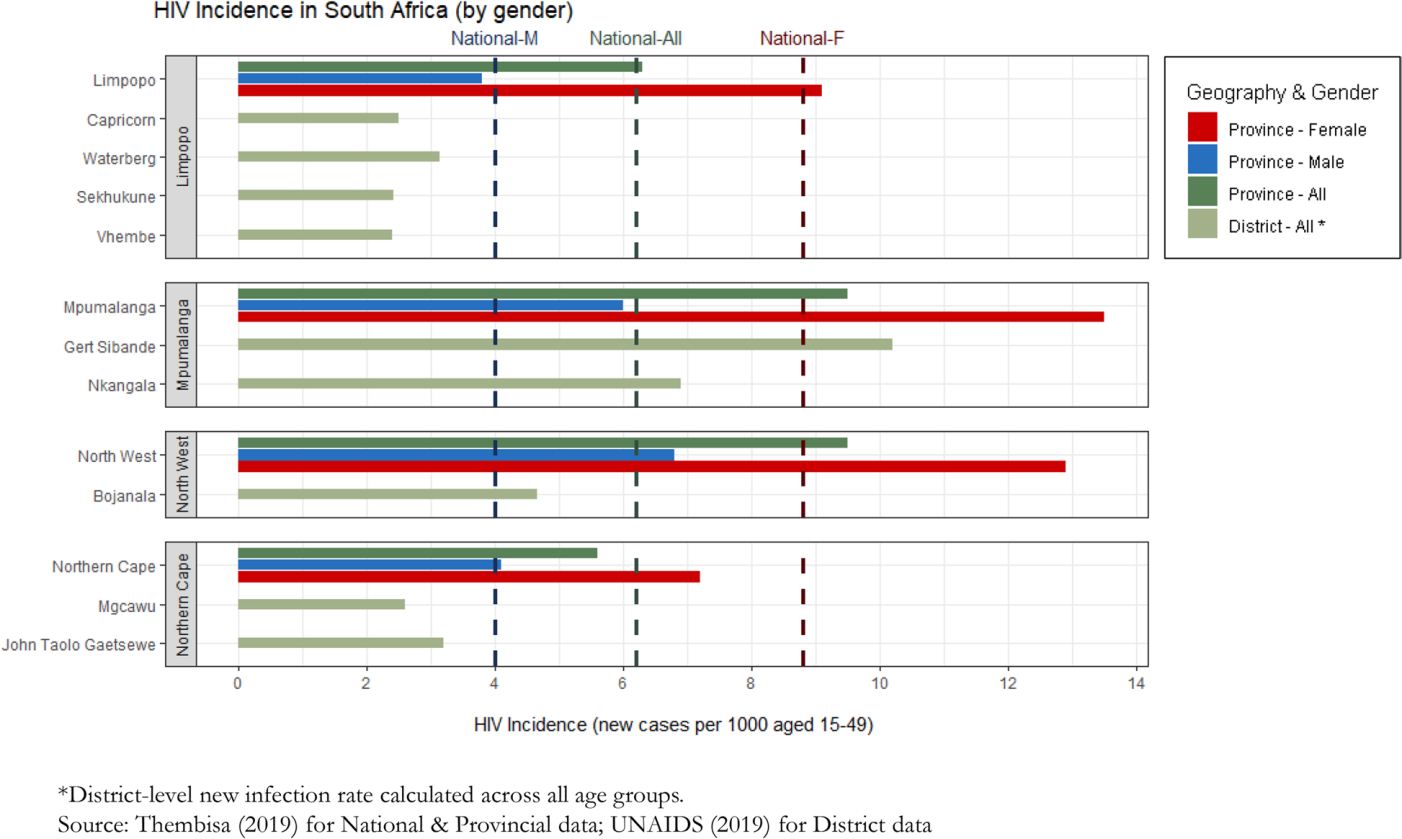
Priority SDG3 indicator 3.3.1 (HIV) by national and sub-national geographies

Based on both the qualitative and quantitative data, the following five indicators under four SDG targets were confirmed as priority areas for action: SDG3.1 (maternal mortality), SDG3.3.1 (HIV), SDG3.3.2 (Tuberculosis), SDG3.5 (Substance Abuse), and SDG3.6 (RTAs). Figures 3 and 4 present the key quantitative data collected against each of these indicators. We now examine the results for each of these priority areas in turn, drawing out key underlying factors.

### SDG priority areas for action

#### Reduce the maternal mortality ratio to < 70 per 100,000 live births and increase the proportion of births attended by skilled health personnel (SDG3.1)

The qualitative appraisal identified maternal mortality as a priority area for action in Greater Tubatse2, Polokwane, and Govan Mbeki. Teenagers and women living with HIV were identified as being particularly vulnerable. Poor access to healthcare, lack of ambulances, shortage of nurses and homebased carers, lack of health promotion activities, and a lack of antenatal care (ANC) equipment were identified as structural factors impacting on maternal mortality. The key community-level factor was insufficient health education, with individual-level factors including women presenting late for ANC (especially teenagers scared to disclose a pregnancy and unable to access services out of school hours), and poor care pathways leading to women delivering at a clinic different to where they access ANC (sometimes due to denial of a HIV diagnosis).

At 138 deaths per 100,000 live births, the burden of maternal mortality in South Africa remains above the SDG target of < 70. There was a particularly high burden of maternal mortality in Polokwane (352.6), eMalahleni (282.6), and Thabazimbi (156.4) municipalities (see Fig. 3a). As compared to the national figure of 96.7%, five districts had a below average percentage of births attended by skilled health personnel (Gert Sibande: 82%; Nkangala: 87.4%; Mgcawu: 67%; John Taolo Gaetsewe: 79.4%; Bojanala: 76.8%).

#### End communicable diseases (SDG3.3)

Participants of the workshops and interviews listed this target as a priority area for action in all but one host community. In Greater Tubatse2, this target was prioritised by key informants but not in the workshop, which consisted of a higher proportion of community members than other workshops. HIV and TB were viewed as the most pressing issues impacting on the health and wellbeing, and undermining economic and social development. In relation to the other diseases explicitly mentioned under SDG3.3, malaria was raised as a consideration at a number of sites, mainly in relation to migrants, but there was no mention of Hepatitis B or neglected tropical diseases. Co-infection of HIV and TB was frequently discussed. Adolescent girls, young women, and female sex workers were viewed as particularly vulnerable to HIV, and there were references to the role alcohol and drug use play in terms of HIV transmission, particularly among these populations.

The annual rate of new HIV infections in all districts was higher than the national average of 6.2 per 1000 uninfected population, which itself is one of the highest new infection rates globally. The HIV data are presented separately in Fig. 4 to indicate that the data are drawn partly from modelled estimates and were collected later. The HIV rate was highest in Gert Sibande at 29.7 new infections per 1000. The annual incidence of TB was above the rate required for TB elimination (in line with SDG targets) in all districts. Rates exceeded the high national burden of 567 cases per 100,000 in Mgcawu, John Taolo Gaetsewe, and Bojanala (see Fig. 3b).

Key structural factors identified as influencing HIV and TB transmission were poor access to quality healthcare and inconsistent access to medication. In-migration was viewed by participants as placing an extra burden on health services. Key societal factors for both infections included poverty, unemployment and food insecurity (affecting treatment adherence). In addition, for HIV, school dropout and a lack of training and recreational facilities for youth were raised, whereas in relation to TB, poor housing, air quality, and ventilation in clinics, transport, and homes were put forward. A key community-level factor was stigma and discrimination for both HIV and TB. Another was socio-cultural and religious beliefs that conflict with prevention, testing and treatment messages (for example, bewitchment; herbal treatment and cures). Interpersonal factors mainly related to HIV, including gender inequalities and gender-based violence, both of which undermine a woman’s ability to utilise prevention methods and increases their vulnerability to HIV exposure. At an individual level, a key issue for TB was access to health education in local South African languages.

#### Strengthen prevention and treatment of substance abuse disorder (SDG3.5)

The qualitative assessment found target 3.5 to be a priority area for action in all sites. It was perceived as a pervasive and growing problem that required urgent response, with young people seen as particularly vulnerable. Alcohol was reported as the most common substance of harmful use or misuse, followed by cannabis (which is decriminalised), compounds mixed with pharmaceutical products such as methaqualone (a sedative), codeine, and Nyaope (heroin, often mixed with ARVs). Throughout our qualitative assessment there was little discussion about mental health and suicide (referred to directly under SDG3.4 and 3.5). The little discussion there was, acknowledged the need to address addiction as a mental health issue and the need to address the mental health needs of marginalised groups such as LGBTIQA+.

Substance use was acknowledged as impacting on SDG3.3 in terms of engaging in high risk sexual activity and hence increased risk of HIV transmission, and on SDG3.6 in terms of being a leading cause of RTAs. Substance use was also perceived to impact on school dropout, child neglect, teenage pregnancy, crime, gang involvement and the perpetration of gender-based violence.

In line with the qualitative results, quantitative evaluation of alcohol consumption in South Africa demonstrated a high average consumption in all provinces under study, as well as a strong gender divide (see Fig. 3c). With the exception of alcohol consumption in women in Limpopo province (1.5 l of pure alcohol consumed per capita), consumption was higher than the national average for women (2.7 L) and men (16.2 L) in all provinces (see Fig. 3c). Substance abuse data mainly related to alcohol, and there was no data on the coverage of treatment facilities (an SDG3.5 indicator).

Structural and societal factors influencing substance use overlapped with those influencing HIV and TB, for example, poor access to treatment services, poverty, unemployment, and a lack of youth facilities. Specific to substance use, a lack of law enforcement relating to alcohol policies and illicit drugs was raised. Community factors included stigma (reducing likelihood of accessing support for drug dependence) and socio-cultural norms that glamorise alcohol and drug use. Interpersonal factors included gender-based violence in households headed by people with alcohol or substance use disorder, and children in violent households being vulnerable to harmful substance use.

#### Reduce the number of global deaths and injuries from road traffic accidents (SDG3.6)

This target was listed as a priority area for action by workshop and / or key informants in ten sites (Blouberg, Mogalakwena, Greater Tubatse, Steve Tshwete, Sishen, Thabazimbi, Govan Mbeki, Tsantsabane, and Rustenburg). Road traffic accidents were strongly associated with concerns about alcohol and drug use. While local-level data was not available, the annual death rate from RTAs was higher (> 21 deaths per 100,000 population) in all four provinces than the national average of 15.9 (see Fig. 3d).

The two key structural factors raised in relation to this target were poor road quality and a lack of traffic and pedestrian safety measures, such as road barriers. Poverty was the key societal factor, with a lack of efficient and affordable private transport (petrol costs) and public transport resulting in a reliance on taxis, whose drivers were deemed to practice poor and risky driving. Expanding the network of ‘scholar patrols’ who help children cross nearby roads to all schools was seen as a priority. At a community level, social norms relating to masculinity were seen to promote poor driving practices such as driving whilst under the influence of alcohol and drugs and speeding, and of being permissive of road rage. Limited first aid experience among community members was equated to a lack of knowledge as to how to stabilise victims of accidents. At an individual-level, poor knowledge of road safety measures, such as driving with lights or walking with reflective clothing, as well as behavioural factors, such as driving impatiently, when tired, and/or when under the influence of alcohol or drugs were raised as concerns.

## Discussion

Applying a mixed-methods approach, we identified local health needs and prioritised SDG3 targets for intervention in host communities of mining operations in South Africa. We found good alignment between priority areas identified qualitatively by stakeholders and the quantitative data collected. Key priority areas for action identified through our alignment of data were SDG3.1 (maternal mortality), SDG3.3 (communicable disease; namely HIV and TB), SDG3.5 (substance abuse) and SDG3.6 (RTAs).

We highlight consistency in the individual, interpersonal, community, societal, and structural factors reported as underlying these targets. At a structural level, poor access to quality healthcare was raised at every workshop as the key factor underlying the achievement of all SDG3 targets. Although most of the priorities we identified impact at the general population level, key populations were identified as being particularly vulnerable, including adolescent girls and young women, female sex workers and LGBTQIA+ populations. Men were identified as a target group in relation to SDG3.5 and SDG3.6 and the need for increased testing among men for communicable diseases was also evident in the discussions.

Considering progress across South Africa, the National Statistical Service states there has been notable progress in reducing the maternal mortality ratio, the under-5 mortality rate, neonatal mortality rate and the infant mortality rate [13]. Despite reporting a decline in the incidence of HIV and TB, they go on to suggest that the incidence of these two infections remains very high and that there has been an increase in the harmful use of alcohol [13].

.Of the five priority areas we identified, HIV, TB and substance abuse were found to overlap in the study communities in terms of risk, burden, and underlying factors. Depending on the extent and type of health, psychosocial and structural interactions, an optimal response may involve targeting potentially linked health-damaging conditions sequentially, or to target one with the expectation of observing collateral effects in other untreated domains of health and wellbeing [14]. To inform a potential coordinated public health intervention approach, we plan to test the extent of interaction across these three conditions and confirm the existence and operational features of a potential syndemic we have defined as SATHA (Substance Abuse; TB; HIV/AIDS). Syndemics are defined as the concentration of two or more diseases or health-damaging conditions in a population, in which there is some level of deleterious biological or behavioural interaction that exacerbates the negative overall burden of any or all the conditions involved [15]. To confirm the syndemic nature of SATHA, we intend to conduct a further review of the literature and also conduct qualitative assessments based on a multi-criteria decision matrix that encompasses the key defining criteria of a syndemic.

Our study had several limitations. Accurately measuring progress towards SDG3 targets was, at times, challenging due to the design of the targets and indicators. For example, the indicator for alcohol abuse relates to pure alcohol per capita, which may not meaningfully capture the full extent to which harmful substance use is occurring and affecting community health. Also, the SDG sub-target to halve deaths resulting from RTAs fails to take into account associated morbidity. As our criteria for community inclusion was distance from a mine, we did not consider potential demographic differences between communities. A recent study that mapped and classified mining host communities in South Africa, adopted four community categories based on population size and level of mining influence [6]. It is possible that differences identified in local health needs may reflect population level differences between communities. Another limitation was that quantitative data were often scarce at the municipal level, and unavailable at the level of host community. In particular, there was relatively little data available on outcomes not directly related to mortality within the health system; this was particularly the case for air pollution. This may reflect priorities of the global health community, which focuses on commonly studied outcomes, such as mortality, that can be routinely collected by health systems.

The authors of a review of under-five mortality in South Africa concluded there were data discrepancies across systems, particularly at the sub-national level, and that action was needed to not only improve data completeness and accuracy but also to strengthen data reconciliation and triangulation [16]. To reduce the impact of limited data in our study, we triangulated data from multiple sources and at different geographical levels. For example, in circumstances where quantitative data on substance abuse were not available at the municipal level (only being available at the level of province), qualitative findings provided key contributions to fill the data gaps. This solution to filling such data gaps, we argue, reinforces the value of a mixed-method approach to baseline health assessment.

The data-powered drive to tackle COVID-19 may present opportunities to address wider deficiencies in the availability of quantitative data. A recent review of existing frameworks for SDG implementation in three cities in the global south, including Cape Town in South Africa, found the link between existing data systems and the data required to monitor and report on SDG implementation is still emerging [17]. The authors of the review suggest that local responses to COVID-19 highlight that required data systems can be put in place quickly.

## Conclusions

Having formed an independent academic consortium to work with a mining sector social-performance team, we successfully demonstrate a mixed method approach to identifying local level priorities as perceived by local stakeholders in line with the SDG3 targets. The consistency in reporting of the priority SDG3 targets across our study communities suggests the need for effective, efficient and feasible interventions to address maternal mortality, HIV, TB, substance abuse, and RTAs. It is essential that these interventions tackle associated upstream factors, including poor access to quality healthcare and medication, as well as societal level factors, including socio-economic determinants of ill-health such as poverty and insufficient education. At a community level there is a need to tackle gaps in knowledge that undermine health seeking behaviour, negative socio-cultural and religious norms, and stigma that continues to impede testing and health seeking behaviours. Interventions also need to be designed to target specific key populations, including adolescents and young people, among whom there is a need to increase recreational and training opportunities. Interactions between the prioritised SDG targets need to be considered when developing interventions.

While there remains considerable debate as to the wider role and ethics of the extractive industries in health, there also remains a clear imperative to urgently address the SDG3 priority targets identified by this study. As long as the mining sector remains present in South Africa, its investment into health may form a crucial part of implementing programmatic activities for achieving the SDG3 health goals.

## Data Availability

The datasets used and/or analysed during the current study available from the corresponding author on reasonable request.

## Abbreviations

SDG: Sustainable Development Goals
RTA: Road Traffic Accidents;
NCD: Non-Communicable Disease
UHC: Universal Health Coverage
SRH: Sexual and Reproductive Health
MMR: Maternal Mortality Ratio
TB: Tuberculosis
HIV: Human Immunodeficiency Virus

## Footnotes

1 These indicators are: SDG3.1.1 (Maternal Mortality Ratio); SDG3.1.2 (Skilled Birth Attendance); SDG3.2.1 (Under 5 Mortality); SDG3.2.2 (Neonatal Mortality); SDG3.3.1 (HIV incidence); SDG3.3.2 (TB incidence); SDG3.4.1 (NCD mortality); SDG3.5.2 (Alcohol Consumption); SDG3.6.1 (RTA mortality); SDG3.7.1 (Family Planning Access); SDG3.7.2 (Adolescent Birth Rate).
